# Phytochemical composition and subacute toxicity of ethanolic extract of *Eucheuma denticulatum* on male Wistar albino rats

**DOI:** 10.1016/j.toxrep.2026.102268

**Published:** 2026-05-07

**Authors:** Mary Muze, Emmanuel Abraham Mpolya, Clara Mollay

**Affiliations:** a The Nelson Mandela African Institution of Science and Technology (NM-AIST), School of Life Sciences and Bioengineering, Arusha, Tanzania; b The Nelson Mandela African Institution of Science and Technology (NM-AIST), Department of Global Health and Bio-Medical Sciences, Arusha, Tanzania; c The University of Dodoma (UDOM), School of Nursing and Public Health, Department of Public Health, Tanzania

**Keywords:** *Eucheuma denticulatum*, Seaweed, Subacute, Oral toxicity, *Ethanolic extract*, *FTIR*, *Tanzania*

## Abstract

*Eucheuma denticulatum* is a rich source of secondary metabolites with established medicinal importance, including its potential use in diabetes management. The current study was conducted to determine the subacute toxicity and the FTIR and LC-MS analysis of the ethanolic extract of *E. denticulatum* following repeated exposure, which is of greater therapeutic value. The crude extract of *E. denticulatum* was prepared using ethanol and administered orally to 4 groups, with each group containing 6 male rats at doses of 300, 600, and 1000 mg/kg, in accordance with the guidelines of the Organization for Economic Cooperation and Development (OECD) (407) over 28 days. Following this, hematological, biochemical, histopathological, and organ and animal weight parameters were examined to assess the toxicological effects of the macroalgae extract. The macroalgae extract did not induce any significant differences in biochemical parameters or hematological results (P > 0.05). However, basophils, hematocrit, and mean corpuscular hemoglobin concentration showed only slight statistical differences in a dose-dependent manner, and all values remained within normal acceptable ranges. The maximum administered dosage showed slight morphological alterations in the kidneys and liver. Body weights in the treatment groups showed time-dependent increases comparable to those of the control group; however, there were no significant variations in organ weights. The FTIR analysis revealed the presence of polyphenols, flavonoids, amides, carboxylic acids, and esters, and LC-MS identified potential biological antidiabetic compounds such as phytol, hexadecenoic acid, tetradecanoic acid, and Eicosapentaenoic acid (EPA). Therefore, preventive steps must be considered when using the *E. denticulatum* extract as a long-term therapeutic application, especially at large doses of 1000 mg/kg b.w.

## Highlights

• The *E. denticulatum* main functional groups in the FTIR analysis.
• After repeated oral dosage for 28 days, subacute toxicity was assessed.
• At tested dose levels, rats showed no discernible harm except at the highest dose.
• There were no significant physical changes upon administration of *E. denticulatum* extract.
• In test animals, the extract showed a good safety profile in the low and middle doses

## Graphical abstract

**Figure:**
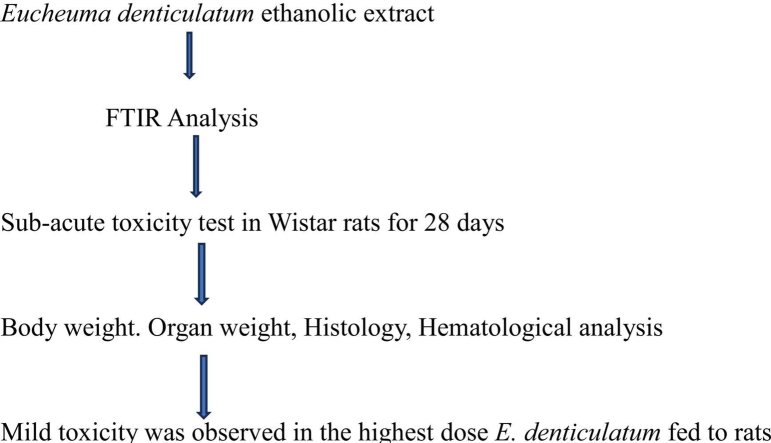

## 1 Introduction

Natural products made from plants have gained interest all over the world, leading to over 80% of people relying on them as a primary source of disease treatment, including diabetes [1]. These natural products include seaweeds that are widely consumed worldwide in the management of diabetes [2], [3]. These natural products play a crucial role in primary healthcare in many developing countries, including Tanzania, where 60% of urban and more than 80% of rural populations depend on traditional therapies for their healthcare services [4]. However, the potential toxicological effect has not been properly assessed. Some studies have documented cases where plants have shown to be toxic when administered either once or repeatedly [5], [6]. Therefore, toxicological evaluations of algae extracts are essential to determining their safety for therapeutic uses.

In 1970, seaweed was imported to Tanzania from the Philippines, with the importation of Euchematoids, commercial seaweed cultivation started in 1989 [7]. It entailed the cultivation of two red macroalgae species that were brought from the Philippines, namely *Eucheuma denticulatum,* also referred to as "spinosum", and *Kappaphycus alvarezii*, also known as "cottonii” [7]. Tanzania started exporting seaweed on a commercial scale in the early 1990's, and currently it is the third-largest industry in the Zanzibar islands, which are located east of Tanzania's mainland [7], [8]. Seaweed has the potential to be a significant source of food and revenue for Tanzanian farmers and can be consumed either fresh, fermented, dried, frozen, whole, powdered, or granulated [9].

Red (Rhodophyta) algae are among the different available seaweeds currently available in Tanzania’s oligotrophic waters along the Western Indian Ocean [7]. It has currently grown and is widely cultivated in Zanzibar, Bagamoyo, Mtwara, Kilwa, and Tanga. Among the available species in Tanzania, the red algae *E. denticulatum* is widely cultivated due to its climate resilience and economic importance [10]. The specie is composed of branches that resemble spines and have thin to sharp points, while the branches are heavily covered in branchlets that range in length from 1 to 8 mm [7]. The algae have been reported to contain phytocompounds that include a variety of interesting bioactivities, including anti-inflammatory, anti-diabetic, anti-obesity, and anti-cancer qualities [11], [12], [13], [14]. Notably, these compounds identified include carotenoids (including zeaxanthin, lutein, and β-carotene), polyphenols (like flavonoids and tannins), and other bioactive substances like carrageenan, which are among the compounds found in this seaweed [2], [3]. Another study by [11] reported that the presence of bioactive compounds such as flavonoids and saponins in *E. denticulatum* has been linked to improved insulin sensitivity and reduced insulin resistance, which are critical aspects in type 2 diabetes.

Some studies have assessed the *E.denticulatum* acute toxicity profile on oral exposure in animal models such as rats [11], [15], indicating that following a single dosage oral exposure of *E. denticulatum,* the estimated LD50 was greater than 2000 mg/kg. However, establishing a strong, comprehensive toxicity profile requires more than one oral dosage exposure to the substance. To establish its safety, a 28-day repeated dosage study is regarded as a thorough toxicological assessment [5], [6]. Therefore, data gathered from the published acute toxicity studies served as a reference for choosing the dosage levels for this investigation. To assess the subacute toxicity of *E. denticulatum* in rats, this study was conducted in light of the numerous established therapeutic advantages of the algae.

## 2 Materials and methods

### 2.1 Algae collection and ethanolic extract preparation

Red seaweed macroalgae *E. denticulatum* species were obtained from Fumba village in Unguja, Zanzibar, on March 28^th,^ 2025, and identified by a botany taxonomist, Gabriel Mwigune, from the Tanzania Plant Health and Pesticides Authority (TPHPA). The identified macroalgae were deposited in the herbarium at TPHPA in Arusha with voucher specimen number Muze, M.001. Following the protocol by [12]. The collected *E. denticulatum* obtained from Fumba village, Zanzibar, were transported to the University of Dar es Salaam in cool boxes at 0 to −4 °C. It was then washed with distilled water to remove dirt and later pulverized into tiny particles. About 2100 mLs of ethanol was used to extract 1000 g of fresh *E. denticulatum*, followed by stirring for 24 h at room temperature. Followed by sonication for 30 min, then filtered using Whatman No. 1 paper. The filtrate was concentrated at a temperature lower than 55 °C in a rotary evaporator. The concentrated extracts were then freeze-dried and stored at −4 °C for further analysis.

### 2.2 Fourier transform infrared spectroscopy and gas chromatography analyses

The functional and phytochemical composition of the *E. denticulatum* crude extract was characterized using an ATR-FTIR Spirit-T Shimadzu spectrophotometer (SN: A224159). The FT-IR spectra of the functional groups were recorded in the scanning range of 4400–400 cm^−1^.

### 2.3 Liquid chromatography- mass spectrophotometry

The tandem TSQ Quantis MS equipment and Thermo-Scientific Vanguish Flex Ultra-High Performance Liquid Chromatography were used to analyse the algae extracts. After dissolving dried crude extracts in methanol to a concentration of 0.25 mg/mL, the mixture was filtered through a 0.22 μm polytetrafluoroethylene syringe filter. The LC-MS apparatus was filled with a 2 μL aliquot of the solution. A Thermo-Scientific Zorbax Eclipse Plus reversed-phase column (100 mm × 2.1 mm, 1.7 μm particle size) kept at 40 °C was used for chromatographic separation. The mobile phase was a binary solvent system. Water with 0.1% formic acid made up mobile phase A, while acetonitrile with 0.1% formic acid will make up mobile phase B. The elution gradient began at 5% B, kept there for two minutes, then climbed linearly to 95% B over ten minutes, stayed there for sixteen minutes, and finally re-equilibrated to the starting conditions between sixteen and twenty minutes. A flow rate of 0.3 mL/min was used. The electrospray ionization source used automated switching to operate in both positive and negative ionization modes during mass spectrometry. First quadrupole mass analyser (Q1) resolution of 0.7, spray voltage of 3700 V (positive) and 2500 V (negative), ion transfer tube temperature of 325 °C, vaporizer temperature of 350 °C, sheath gas flow of 10 L/min, and auxiliary gas flow of 5 L/min were the source parameters. Thermo Scientific TraceFinder 7.2 software was used to collect the data. MS/MS used the automated. Multiple Reaction Monitoring mode with a 6 eV collision energy. Replicate injections were used to assess the LC-MS performance, and the peak regions and retention times were produced in triplicate. The National Institute of Standards and Technology Library's 2021 spectral database was used to identify compounds, and the confidence was through possible identification based on fragmentation pattern and spectral library match, and tentative identification based only on mass spectral data.

### 2.4 Experimental animals (source, sex, weight, and housing)

About 24 male Wistar albino rats, weighing 110–119 g, were used in this sub-acute toxicity study obtained from the Faculty of Veterinary at Sokoine University of Agriculture, Morogoro, Tanzania. Standard housing conditions were (25 ± 2 °C, relative humidity 45–55%, and 12-h light/dark cycle) and had free access to food and water *ad libitum*. Male Wistar rats were selected due to their higher sensitivity [16]. The Tanzania Animal Welfare Act, the guidance for the care and use of laboratory animals, and other national and international ethical guidelines for animal rights were used in handling the animals. The procedures involving animals were approved by the Nelson Mandela African Institution of Science and Technology and the National Institute for Medical Research, with reference number NIMR/HQ/R.8a/Vol.IX/4951.

### 2.5 Sub-acute toxicity testing (oral repeated 28-day exposure)

This study was conducted in accordance with the OECD Guideline (407), with minor changes to the use of single-sex male rats for a 28-day repeated oral exposure toxicity test. A total of twenty-four male rats were used due to their documented high sensitivity in subacute toxicity studies [17]. Based on previous studies of the acute toxicity of the macroalgae, the doses selected for subacute oral administration were 300, 600, and 1000 mg/kg. These doses were selected based on safety and pharmacological activity. The acute toxicity test of *E. denticulatum* ethanolic extract was found to be safe at dosages below 2000 mg/kg b.w [11] and the OECD guideline on the selection of the highest dose of 1000 mg/kg b.w was selected since it's half of the limit dose, and (600, 300) mg/kg b.w were the intermediate dose and low dose, providing a dose relationship, and these doses were the effective pharmacological antidiabetic activity.

This study grouped the rats into four with each group containing 6 rats. Group 1 received distilled water while groups 2–4 received a solution of *E. denticulatum* extract at the doses of 300, 600, and 1000 mg/kg, respectively, for 28 days. The administration of the *E. denticulatum* extract was done every morning. On the 29th day, the rats were sacrificed by cervical dislocation using 3% pentobarbital sodium after fasting for about 12 h. A deep longitudinal incision was made into the ventral surface of the abdomen and thorax of the sacrificed rats. By blunt dissection of the muscles and fasciae, vital organs, including the liver, kidneys, heart, and adipose tissue, were exposed and collected.

### 2.6 Measurement of body weight

The baseline body weight of the individual animals was recorded on day 0 before the experiment began and on days 7, 14, 21, and 28 of the experiment.

### 2.7 Hematological analysis

Hematological analysis was conducted by collecting 2 mL of the blood and placing it in a Vacutainer tube with EDTA. Then, hematological analysis was performed using a Veterinary automated hematology analyzer. The aforementioned analyzed the red blood cells (RBC), White blood cells (WBC), lymphocytes (Lym), monocytes (Mon), basophils (Bas), hemoglobin (Hb), hematocrit (Hct), mean corpuscular volume (MCV), mean corpuscular hemoglobin concentration (MCHC), mean corpuscular hemoglobin (MCH), red cell distribution width (RDW), mean platelet volume (MPV), procalcitonin (PCT), and platelet distribution width (PDW).

### 2.8 Biochemical analysis

About 2mls of the blood taken into the plane vacuum container tubes were subjected to a biochemical parameter analysis. To obtain serum, the blood tubes were centrifuged for 5 min at 10,000 rpm. A UV/Visible Spectrophotometer was used to analyze the biochemical parameters of the acquired serum. The analysis was done using kits from Erba Mannheim, Germany. Therefore, analysis of aspartate aminotransferase (AST), alanine aminotransferase (ALT), total protein, creatinine, and urea was examined.

### 2.9 Histopathological analysis

The liver, kidney, heart, and adipose tissues of each rat were gently collected and fixed in 10% neutral buffered formalin. Small Section (4 mm thick) from each tissue were cut and processed for histopathological analysis of liver, kidney, heart, and adipose tissue. The first procedure was dehydrating the tissue in increasing ethanol concentrations (70%, 90%, 95% and 100%), followed by clearing with chloroform, then infiltrating with tissue paraffin wax, and lastly embedding in a melted tissue paraffin wax to form an embedded tissue block for sectioning. The tissue ribbons were sectioned from the embedded tissue blocks at 3μm using a microtome machine. Sectioned tissue ribbons were transferred into cold water, then into a hot water bath (45 ◦C), and then mounted on a cleaned microscope slide and lastly dried in a hot air oven for 12 h. Tissue sections were deparaffinized in three changes of xylene followed by rehydration through a descending series of ethanol concentration (100%, 95%, 90% and 70%), to distilled water. The sections were stained in Haematoxylin solution for 4 min, washed in distilled water, then differentiated in 1% acid – alcohol for a few seconds and later washed again in distilled water. The tissues were blued in an alkaline (lithium carbonate) solution and washed in distilled water. Later, the tissues were counterstained with eosin solution for 5 min, dehydrated in methanol, cleared in xylene, and mounted on a microscope glass coverslip using mounting solution (dibutylphthalate polystyrene xylene, DPX). Stained tissues were visualized, measured, and photographed on an Olympus Light Microscope (Olympus Corporation, Model D21-CB, SN 0010842A2, Tokyo, Japan) equipped with an adjusted digital camera (Olympus U-TV0.5XC-3, SN OK73198, Tokyo, Japan). All images for histomorphometric analysis were made in μm, at × 40 and 400 magnification.

## 3 Statistical analysis

The mean ± standard error of the mean was used in the representation of the data. Genstat 15th Edition software was used in the analysis of data. The differences across the control and treatment groups were analyzed using one-way analysis of variance (ANOVA), and for any significant differences that were detected, Dunnett’s post hoc test was used to compare the control and treatment groups. A p-value of p < 0.05 at 95% confidence level, the results were considered statistically significant.

## 4 Results

### 4.1 LC-MS AND FTIR of the *E. denticulatum* extracts

**Fig. 1:**
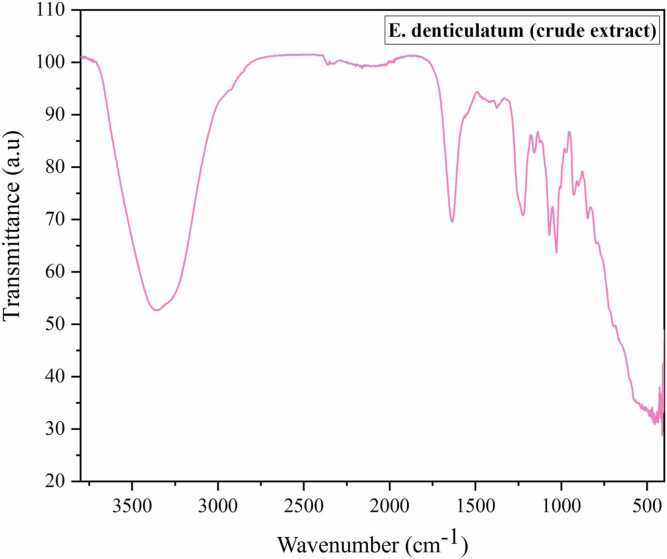
FTIR spectrum of the crude *E. denticulatum* extract.

**Fig. 2:**
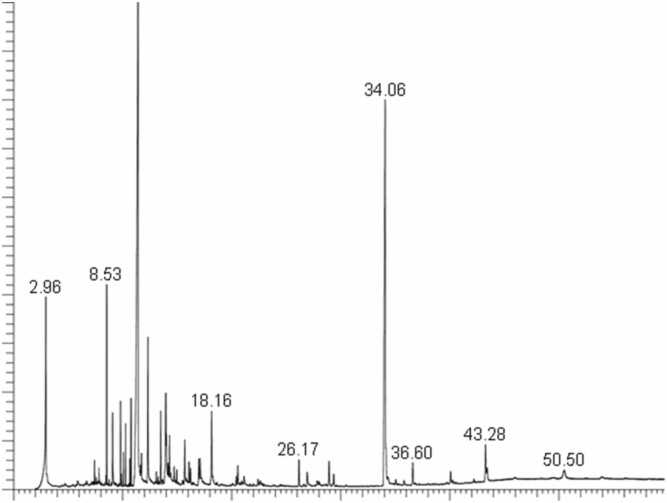
LC-MS chromatogram of ethanolic extract of E. denticulatum.

The FTIR spectrum of the current study revealed vibrational frequencies at O-H bond: 3300, C-N: 1200, C=O: 1600, C-OH: 1050, indicating the presence of polyphenols, flavonoids, esters, carboxylic acid, and amides in *E. denticulatum* extract (Fig. 1). The composition of the extract of *E. denticulatum* was analyzed using a liquid chromatography-mass spectrophotometer (LC-MS). In the current investigation, LC-MS analysis was performed on the ethanolic extract of *E. denticulatum*. The LC-MS results showed that fifty-nine bioactive compounds were present (Table 1). Additionally, the results revealed the molecular formulae, molecular weights, compositions (%), and retention times determined from the peak areas of each compound (Fig. 2**).**

**Table 1:** FTIR peak identification of *E. denticulatum*.

FT-IR Region (cm⁻¹)	Functional Group	Compound Class Indicated
3200–3500	O–H	Polyphenols
2920–2850	C–H	Lipids, terpenoids
1650–1600	C=O / C=C	Flavonoids
1540–1450	N–H / C–H	Proteins
1200–1000	C–O / C–O–C	Polysaccharides
900–700	Aromatic / sulfate	Carrageenan

Source: [18], [19]

### 4.2 Evaluation of food, water intake, mortality, and clinical signs

No mortality was observed among the experimental rats. Rats in the control and treatment groups showed identical behavior in terms of locomotor activity, food intake, and water intake. Clinical signs, such as fur, tremors, convulsions, and salivation, were observed in the control and experimental groups (Table 2).

**Table 2:** Results of the evaluation of food and water intake, mortality, and clinical signs.

Physical signs	Tremors	Convulsions	Salivation	Respiration	Fur	Lethargy	Food intake	Water intake	Locomotion	Death
Control	N	NS	N	N	N	NS	N	N	N	NS
300 mg/kg *E. denticulatum* 600 mg/kg *E. denticulatum* 1000 mg/kg *E. denticulatum*	NS NS NS	NS NS NS	N N N	N N N	N N N	NS NS NS	N N N	N N N	N N N	NS NS NS

Whereas N: Normal; NS: Not Seen

### 4.3 Effect of *E. denticulatum* extracts on the body weight of Wistar rats

**Fig. 3:**
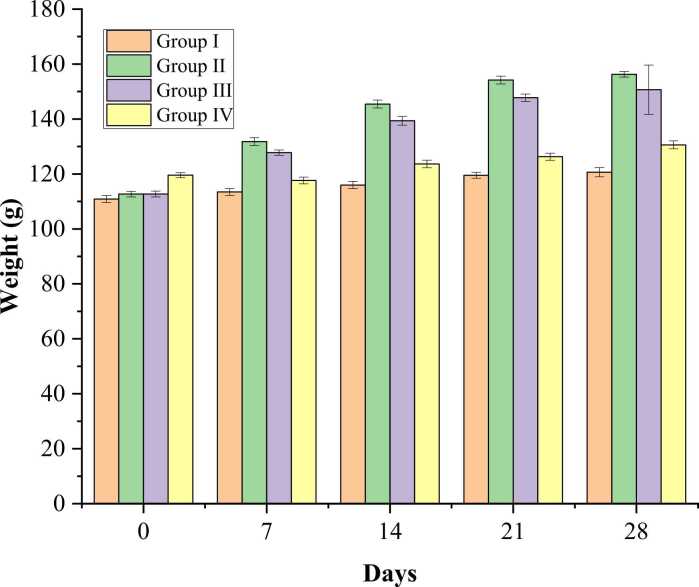
Body weight of experimental Wistar rats on a weekly basis. Group I: Stands for normal control. Group II: 1000 mg/kg of b.w (High dose) of *E. denticulatum.* Group III: 600 mg/kg of b.w (Medium dose) of *E. denticulatum.* Group IV: 300 mg/kg b.w (Low dose) of *E. denticulatum.*

The initial body weight of Wistar rats ranged from 110 g to 119 g in the experimental groups, as shown in Fig. 3. Group I stands for normal control, which exhibited 8.88% increase in body weight gain from day 0 to day 28. Group II, which stands for a high dose of 1000 mg/kg b.w, exhibited a 38.75% increase in body weight from day 0 to day 28. Group III, which stands for medium dose of 600 mg/kg b.w, exhibited an increase of 33.60% increase in body weight from day 0 to day 28. Group IV, which stands for low dose 300 mg/kg b.w, exhibited an increase of 9.26% from day 0 to day 28 (Fig. 3).

### 4.4 Effect of *E. denticulatum* extracts on the Organ weight of Wistar rats

**Fig. 4:**
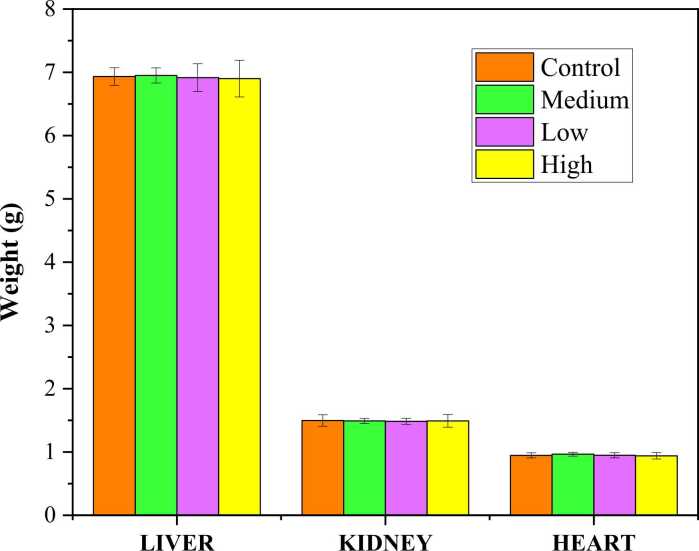
The mean organ weight of experimental Wistar rats at day 29. Group I: Stands for normal control. Group II: 1000 mg/kg of b.w (High dose) of *E. denticulatum.* Group III: 600 mg/kg of b.w (Medium dose) of *E. denticulatum.* Group IV: 300 mg/kg b.w (Low dose) of *E. denticulatum.*

The weights of important organs, including the liver, kidney, and heart, were measured and are presented in (Fig. 4). The organ weights of the treated and control groups did not differ significantly at p-value > 0.05.

### 4.5 Effect of *E. denticulatum* extract on biochemical parameters

The biomarkers from the blood that represent liver and kidney toxicity are presented in (Table 3). The analysis of the blood biochemical parameters showed no significant difference in the parameters, including creatine (CRET), total protein (TP), alanine aminotransferase (ALT), and urea, between the treatment groups and the control at p-value > 0.05 (Table 3). Interestingly, at the highest dose (1000 mg/kg body weight), aspartate aminotransferase demonstrated a significant decrease in comparison to the control at p-value < 0.05 (Table 3).

**Table 3:** Results of the evaluation of biochemical parameters on day 28.

**Parameters**	**Standard values**	**Control group**	**Group I** **(1000 mg/kg b.w)**	**Group II** **(600 mg/kg b.w)**	**Group III** **(300 mg/kg b.w)**
Blood Urea Nitrogen (mg/dl)	15–29	22.73 ± 1.27	25.07 ± 1.37	22.98 ± 1.97	24.77 ± 1.30
Total protein (g/dl)	6.3–8.7	6.48 ± 0.16	5.79 ± 0.47	5.26 ± 0.17	6.30 ± 0.43
Creatinine (mg/dl)	0.9–1.5	1.08 ± 0.32	0.56 ± 0.05	0.56 ± 0.05	0.64 ± 0.05
AST (U/L)	37–92	91.39 ± 2.87	88.44 ± 4.00	81.23 ± 3.54	74.41 ± 3.70*
ALT (U/L)	17–50	36.18 ± 1.91	26.18 ± 3.08	23.8 ± 2.02	30.46 ± 3.93

Values are significantly different from the control group at *p < 0.05, Dunnett’s Multiple Comparison Test, all values are expressed as mean ± SEM for 6 Wistar rats in each group.

### 4.6 Effect of *E. denticulatum* extract on hematological parameters

There were no significant differences in the hematological parameters of the *E. denticulatum* extract-treated groups as compared to the control group, except for the Hematocrit, Basophils, and the mean corpuscular hemoglobin concentration, as presented in (Table 4). However, all values remained within normal acceptable ranges. Thus, the results suggest that *E. denticulatum* had fewer effects on the production of the circulating blood cells due to the extract.

**Table 4:** Effect of *E. denticulatum* administration on hematological parameters at the end of the 28-day treatment period.

SN	Parameter	1000	600	300	Control	Acceptable range values
1	WBC(m/mm3)	6.59 ± 1.43	7.08 ± 1.21	7.09 ± 1.53	7.46 ± 1.08	3.20–10.50
2	Lym(%)	40.26 ± 2.64	40.48 ± 1.73	40.46 ± 4.60	42.06 ± 2.30	65.50–91.70
3	Mon(%)	5.21 ± 0.33	5.36 ± 0.51	5.23 ± 1.55	5.13 ± 0.27	0.22–7.75
4	Neu(%)	5.13 ± 1.51	5.22 ± 1.18	5.54 ± 2.34	5.56 ± 3.15	4.30–24.70
5	Eosinophils (%)	1.50 ± 0.50	1.20 ± 0.21	1.50 ± 0.39	2.63 ± 1.09	0.0–3.90
6	Bas(%)	0.96 ± 0.06*	0.68 ± 0.13	0.31 ± 0.04	0.36 ± 0.13	0.10–5.0
7	RBC(M/mm3)	7.51 ± 0.09	6.18 ± 1.04	7.15 ± 0.47	7.08 ± 0.24	6.73–8.57
8	MCV (ft)	54.36 ± 1.89	54.05 ± 0.76	54.66 ± 0.43	52.28 ± 0.48	45.90–63.96
9	Hct(%)	41.1 ± 0.52*	33.53 ± 2.48	33.01 ± 0.83	33.83 ± 1.55	39.0–51.0
10	MCH (pg)	19.31 ± 0.06	19.61 ± 0.32	19.2 ± 1.36	19.11 ± 0.16	17.05–22.03
11	MCHC(g/dl)	32.01 ± 0.80*	36.33 ± 0.42	35.36 ± 0.23	35.36 ± 0.04	29.38–34.0
12	RDW	14.75 ± 0.13	14.98 ± 0.27	14.83 ± 0.72	15.76 ± 0.28	13.03–16.57
13	Hb(g/dl)	13.2 ± 0.27	13.73 ± 0.40	12.13 ± 0.488	12.75 ± 0.49	8.50–15.38
14	MPV (fl)	5.66 ± 0.10	5.75 ± 0.09	5.91 ± 0.17	5.51 ± 0.10	6.70–8.10
15	Pct(%)	0.29 ± 0.01	0.28 ± 0.01	0.26 ± 0.10	0.33 ± 0.03	0.16–0.35
16	PDW	4.55 ± 0.18	4.51 ± 0.17	4.68 ± 0.14	4.51 ± 0.13	6.75–8.85

One-way ANOVA followed by Dunnett's test was used in the analysis, and the results were presented as mean ± SEM. Differences between groups were considered significant at p < 0.05 in comparison to the control. The acceptable ranges of hematological data were based on [20]

Effect of *E. denticulatum* on Histopathology

## 5 Discussion

The Fourier transform infrared spectroscopy gives a high-quality analytical approach for the determination of the structural compounds in plant extracts. It is a rapid and non-destructive analysis for fingerprinting plant extracts or powders. This analysis revealed the presence of polyphenols, flavonoids, esters, carboxylic acid, and amides. The reported medicinal properties of these plants may be linked to the characteristic functional groups, as illustrated in (Fig. 1) (Table 1), which reports the presence of polyphenols, flavonoids, amides, carboxylic acids, and esters, which supports the claim of the pharmacological and biological activity, including diabetes. Similar findings from this study were reported by [16], [21], that O-H bond: 3300, C-N: 1200, C=O: 1600, C-OH: 1050 of the detected functional groups from the *Urtica dioica* medicinal herb that is used in the treatment of diabetes and other inflammatory diseases.

The advancements in toxicology highlight how crucial it is to evaluate the toxicity of substances to identify potential over a single or repeated exposure. Toxicological models are used to do this, mostly using animal models that are very comparable to humans in terms of their biochemistry, physiology, and pathology [22], [23]. This suggested the use of Wistar rats as a preferred model in this study.

LC-MS analysis of *E. denticulatum* ethanolic extract identified the presence of several phytocompounds that have been reported in the literature with potential antidiabetic biological activity (Table 2) (Fig. 2). The presence of phytol has biological potential to improve insulin sensitivity and aid in the control of hyperglycemia, and reduce elevated blood sugar [24]. It functions as a precursor to phytanic acid, which enhances lipid metabolism and reduces inflammatory markers like TNF-α by binding to and activating peroxisome proliferator-activated receptor gamma (PPARγ) [25]. It is responsible for reducing inflammation and reactive oxygen species that occur as a result of elevated hyperglycemia [25]. Also, it enhances glucose tolerance, lowering body weight, and reducing fat mass in part by blocking enzymes that hydrolyze carbohydrates, like amylase and β-glucosidase [24], [25]. Another compound is Eicosapentaenoic acid (EPA), which has antidiabetic biological activity, mainly by reducing chronic inflammation, increasing insulin sensitivity, and reducing complications from diabetes [26]. Tetradecanoic acid has the biological activity of inhibiting the α-amylase and α-glucosidase enzymes that are responsible for controlling postprandial hyperglycemia in diabetes treatment [27], [28]. The compound inhibits the activity of α-amylase and α-glucosidase enzymes that convert carbohydrates into glucose after meals, which is released into the bloodstream, which is particularly crucial for controlling postprandial hyperglycemia. Finally, the compound Hexadecanoic acid, methyl ester, is also a potential compound with antidiabetic biological properties, since it has been studied to have antioxidant potential, hypocholesterolemic, and alpha reductase inhibitor [27], [29]. However, no pharmacological study has been conducted of the *E. denticulatum* extract to support the claims.

Natural products are widely regarded as safe in developing countries healthcare systems [23], with various studies that have documented their toxicity [30], [31]. It is therefore necessary to perform toxicological studies to acquire information on the safety profile [6], [32]. Therefore, this study is a critical step in determining the macroalgae safety profile and is crucial for future research into its potential as a source of contemporary therapies. Notably, this is the first study to assess the sub-acute toxicity of *E. denticulatum* extract in animal models. The extract administration did not cause any changes in the physical signs or mortality in the rats (Table 3).

Abnormal changes in the animal body and organ weights are the earliest indications of toxicity following repeated exposure to a substance as such, they are regarded as crucial markers for adverse effects [32], [33]. *E. denticulatum* extract given at the doses of 300, 600, and 1000 mg/kg increased the body weight in a concentration-dependent manner (Fig. 3). This increase in body weight may be due to the high protein content and improved protein digestibility due to the building up of lean tissues, and increased repair of tissues. Similar studies on marine algae *Osmundari obtusiloba* where higher body weight has been linked to a nutrient-rich composition [22], [34], [35]. However, the exact mechanism was not elucidated in this study. Nevertheless, there was no discernible difference between the organ weights of the animals given the algae extract and the control group (Fig. 4). According to earlier research, decreased weights often indicate the toxic effect of the tested substance [30] [32]. These results are similar to a study by [36] that indicated the supplementation of dietary tropical seaweed in Wistar rats did not induce any changes in the weight of the organs. Therefore, the results of this study indicate no toxicity to the organ weight.

The *E. denticulatum* extract did not significantly raise any of the biochemical markers that were examined when it was compared to the control group (Table 4). These findings imply that the extract did not alter the biochemical parameters. However, the low AST levels in the lower dose (300 mg/kg b.w) were not dose dependent, which signifies normal variability of the Wistar rats and is supported by histopathological parameters showing no toxicity. The results resemble those of *Gracilaria changii,* conducted by [34], who reported that the seaweed did not cause any toxicity on liver and renal tissues, which might be due to the antioxidant and high fiber content present in the seaweed. Another study by [35], [36] also reported that the red seaweed *Osmundaria obtusiloba,* reported that the seaweed did not cause any significant changes in the biochemical markers.

The hematological system is one of the most vulnerable indicators of toxicity and is also a crucial indicator of both human and animal physiological and pathological conditions [6], [37]. Therefore, hematological parameter evaluations were conducted to determine its impact after repeated exposure to *E. denticulatum* extract (Table 4). Although the majority of hematological indices assessed showed no significant difference compared to the control group, except for hematocrit, mean corpuscular hemoglobin concentration, and basophils, all values remained within normal, acceptable ranges (Table 4). The dose-dependent increase in basophils showed that the ethanolic extract of *E. denticulatum* contains compounds that trigger the immune system through the production of basophils. Conversely, a study by [38] showed that substances found in brown seaweed, like chlorophyl C2 and sargahydroquinoic acid (SHQA), inhibit basophil activation and degranulation. In addition to that, an increase in hematocrit and mean corpuscular hemoglobin concentration could be explained by indicators of overhydration from the high-dose extract, resulting in a concentrated increase in the hematocrit and mean corpuscular hemoglobin. Similar findings were reported by [39], who found in human trials that seaweed may increase mean corpuscular hemoglobin concentration and hematocrit, which is probably due to iron and vitamin content that promotes erythropoiesis, or the creation of red blood cells. Due to their limited magnitude and lack of dose relationship response of the hematological parameters, signifying there were minor variations in the physiology of the Wistar rats. However, since none of the immunological experiments were carried out, specific impacts on immune function cannot be exactly elucidated.

**Fig. 5:**
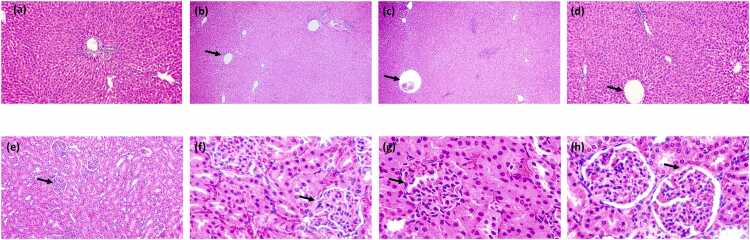
Photomicrograph of the liver and Kidney of rats treated with *Eucheuma denticulatum* (a) Liver section of the rat treated with the 300 mg/kg showing normal histology (Black arrow indicating normal central vein), (b) Liver section of the rat treated with the 600 mg/kg, showing normal histology (Black arrow indicating normal central vein) (c) Liver section of the rat treated with the 1000 mg/kg (Black arrow indicating infiltration of inflammatory cells, congestion of portal vein, hepatocytes and sinusoids are enlarged), (d) Liver section of the rat from the control group showing normal histology (Black arrow indicating normal central vein). (e) Kidney section of the rat treated with the 300 mg/kg showing normal histology (Black arrow indicating normal renal capsule), (f) Kidney section of the rat treated with 600 mg/kg showing normal histology (Black arrow indicating normal renal capsule), (g) Kidney section of the rat treated with the 1000 mg/kg showing histopathology (Black arrow indicating normal bowman’s capsule, dark stained cells or pyknosis, lumens have lost architecture and infiltration of inflammatory cells) (h) Kidney section of the rat from the control group showing normal histology (Black arrow indicating normal renal capsule).

Nonetheless, the most reliable indicators of toxicant-induced effects are thought to be histopathological abnormalities or changes in the tissue organs [6], [30]. The histopathology results of the liver and kidney tissues displayed normal architecture, except for the group treated with the highest dose of the extract (1000 mg/kg b.w) (Fig. 5), which demonstrated slight changes in their morphologies. However, the observed changes in the architecture of the liver showed infiltration of inflammatory cells, congestion of portal vein, hepatocytes and sinusoids are enlarged and the kidney showed bowman’s capsule, dark-stained cells or pyknosis, lumens have lost architecture, and infiltration of inflammatory cells, especially at the maximum dose, were not substantial enough to cause a significant elevation of the biochemical indices, reinforcing the safety of the macroalgae extract. In light of this, individuals should exercise caution while using this macroalgae at maximum dosages, and its extended exposure should be avoided.

The minor histopathological and hematological abnormalities were noted at the highest dose, with implications that alterations did not coincide with significant biochemical disruptions, which suggests organ dysfunction. This lack of accordance of histopathology and biochemical results implies a mild rather than an early toxicity. In addition to that, the low and medium doses lack consistent hematological, biochemical, or histopathological changes, suggesting that the extract was generally well tolerated at low and moderate doses.

## 6 Conclusion

The present study evaluated the phytochemical composition and the toxicological effects of the ethanolic extract of *Eucheuma denticulatum* in Wistar rats. The results showed a total of fifty-nine bioactive and some important bioactive phytocomponents present were phytol, lucenin 2, tetradecanoic acid, hexadecenoic acid, and eicosatetraenoic acid (EPA). The results also indicated that *E. denticulatum* extract tolerated at 300 mg/kg b.w and 600 mg/kg b.w, imparted by the extract on the biochemical, organ weight, hematological, and histopathological parameters, except at the highest doses of 1000 mg/kg b.w. This study also demonstrated an increase in body weight for the treated rats compared to the control group. Given these results, preventive steps must be considered when using the extract as a long-term therapeutic application, especially at large doses of 1000 mg/kg b.w. In this regard, the use of *E. denticulatum* seaweed extracts available in Tanzania should be evaluated for a wide range of biochemical parameters, such as bilirubin, albumin, alkaline phosphatase (ALP), and should also consider quantitative grading of histopathology that includes lesions to support the claims.

## Ethics approval

This study was approved by the National Institute for Medical Research (NIMR) with a reference number NIMR/HQ/R.8a/Vol.IX/4951

## Role of funding source

The funding agency had no involvement in the study design, collection, analysis, and interpretation of data, writing of the manuscript, or the decision to submit the article for publication.

## CRediT authorship contribution statement

**Mary Muze:** Writing – review & editing, Writing – original draft, Investigation, Data curation, Conceptualization. **Emmanuel Abraham Mpolya:** Writing – review & editing, Supervision, Methodology, Conceptualization. **Clara Mollay:** Writing – review & editing, Validation, Supervision, Conceptualization.

## Declaration of Competing Interest

The authors declare that they have no known competing financial interests or personal relationships that could have appeared to influence the work reported in this paper

## Data Availability

Data will be made available on request.
