# Supplementary material for: Phytochemical composition and subacute toxicity of ethanolic extract of Eucheuma denticulatum on male Wistar albino rats
Source: Toxicol Rep. 2026 May 7;16:102268. doi: 10.1016/j.toxrep.2026.102268 (PMC13188117; doi:10.1016/j.toxrep.2026.102268)
Supplement: Supplementary file 1 — Supplementary material [file mmc1.docx]

Table 2. The ethanolic extract of *E. denticulatum* was shown to have major bioactive components.

| No. | Phytochemical Compound | RT  (min) | M.F | M. Wt | Peak  Area% | Chemical structure |
| --- | --- | --- | --- | --- | --- | --- |
| 1 | Ethanol, 2-methoxy-,  acetate (CAS) | 2.95 | C5H10O3 | 118 | 5.51 | 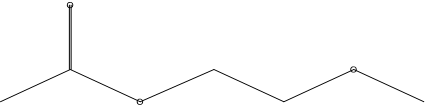 |
| 2 | Retinal | 4.69 | C20H28O | 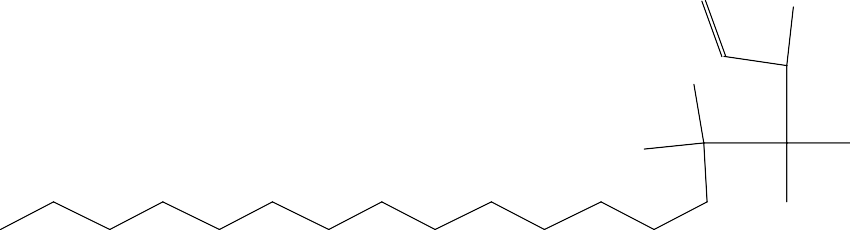284 | 0.16 |  |
| 3 | 2,2,3,3,4,4  HEXADEUTERO | 5.45 | C18H30D6O | 268 | 0.12 |  |
| 4 | Hexadecanoic acid,  phenylmethyl ester | 5.84 | C23H38O2 | 346 | 0.31 | 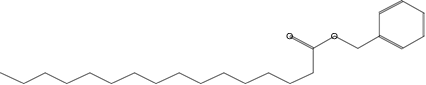 |
| 5 | Octadecanal, 2-bromo- | 6.50 | C18H35BrO | 346 | 0.06 |  |
| 6 | Cholestan-3-ol,  2-methylene-, (3á,5à)- | 6.64 | C₁₄H₃₀O | 214 | 0.20 | 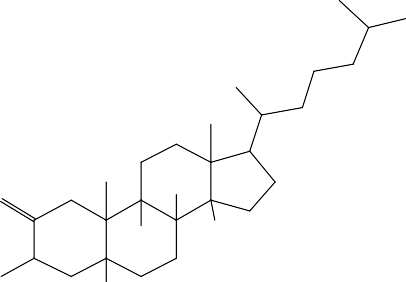 H  H  H  HO  H |
| 7 | 1,3,5-Triazine-2,4-diam  ine, 6-chloro-N-ethyl-  (CAS) | 7.22 | C5H8ClN5 | 173 | 0.06 | 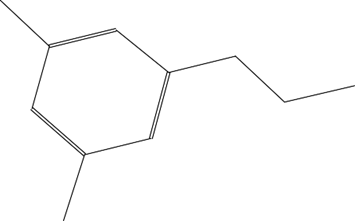 |
| 8 | Dotriacontane (CAS) | 7.31 | C32H66 | 450 | 0.06 | 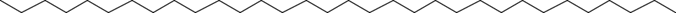 |
| 9 | Albocycline | 7.69 | C18H28O4 | 308 | 0.09 | 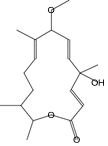 |
| 10 | Phosphonofluoridic  acid, propyl-, nonyl  ester | 7.78 | C12H26FO2P | 252 | 0.42 | 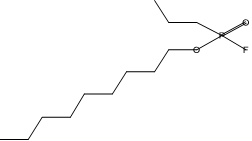 |
| 11 | Baimuxinal | 7.94 | C15H24O2 | 236 | 0.11 | 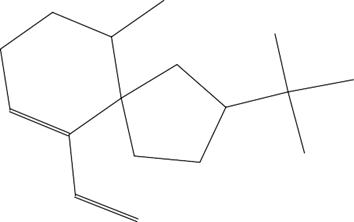 |
| 12 | Lecocarpinolide H | 8.32 | C15H18O5 | 278 | 0.03 | 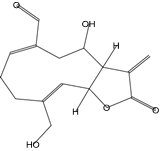 |
| 13 | Nonadecane (CAS) | 8.53 | C19H40 | 268 | 2.90 | 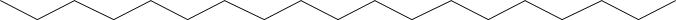 |
| 14 | Tetradecanoic acid,  methyl ester (CAS) | 8.76 | C15H30O2 | 242 | 0.12 |  |
| 15 | 2-Hexadecanol (CAS) | 8.97 | C16H34O | 242 | 0.05 | 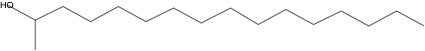 |
| 16 | Tetradecanoic acid  (CAS) | 9.08 | C14H28O2 | 228 | 1.80 |  |
| 17 | 1,3,5-Triazine-2,4-diam  ine, 6-chloro-N-ethyl-  (CAS) | 9.44 | C5H8ClN5 | 173 | 0.05 | 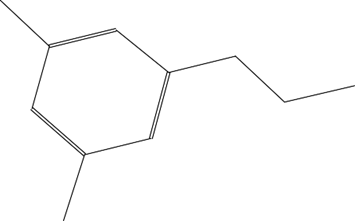 |
| 18 | Ethanol,  2-(9-octadecenyloxy)-, | 9.62 | C20H40O2 | 312 | 0.08 | 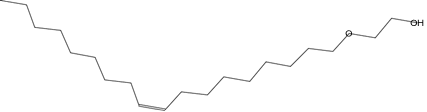 |
| 19 | Neophytadiene | 9.80 | C20H38 | 278 | 1.38 | 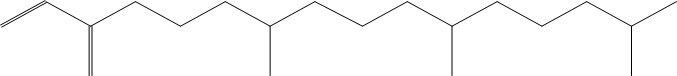 |
| 20 | 2-Pentadecanone,  6,10,14-trimethyl-  (CAS) | 9.89 | C18H36O | 268 | 0.22 |  |
| 21 | 3,7,11,15-Tetramethyl  -2-hexadecen-1-ol | 10.06 | C20H40O | 296 | 0.65 | 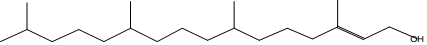 |
| 22 | 9-Hexadecenoic acid,  methyl ester, (Z)-  (CAS) | 10.66 | C17H32O2 | 268 | 0.53 |  |
| 23 | Palmitic Acid methyl  ester | 10.76 | C17H34O2 | 270 | 1.73 |  |
| 24 | (E)-Hexadec-9-enoic  acid | 11.14 | C16H30O2 | 254 | 0.07 |  |
| 25 | Hexadecanoic acid  (CAS) | 11.39 | C16H32O2 | 256 | 26.67 |  |
| 26 | 2-Hexadecen-1-ol,  3,7,11,15-tetramethyl-  , [R-[R*,R*-(E)]]-  (CAS) | 11.66 | C20H40O | 296 | 0.22 |  |
| 27 | Tetradecanoic acid,  12-methyl-, methyl | 11.72 | C16H32O2 | 256 | 0.53 |  |
| 28 | Hexadecanoic acid,  2,3-dihydroxypropyl  ester (CAS) | 12.10 | C19H38O4 | 330 | 0.11 | 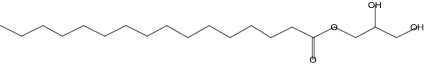 |
| 29 | 9-Octadecenoic acid  (Z)- (CAS) | 12.97 | C18H34O2 | 282 | 0.09 | 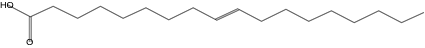 |
| 30 | 2-Hexadecen-1-ol,  3,7,11,15-tetramethyl-  , [R-[R*,R*-(E)]]-  (CAS) | 13.08 | C20H40O | 296 | 0.30 | 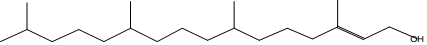 |
| 31 | 10-Octadecenoic acid,  methyl ester (CAS) | 13.25 | C19H36O2 | 296 | 0.15 |  |
| 32 | Phytol | 13.49 | C20H40O | 296 | 1.94 | 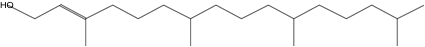 |
| 33 | 8,11,14-Eicosatrienoic  acid, (Z,Z,Z)- (CAS) | 13.67 | C20H34O2 | 306 | 0.22 | 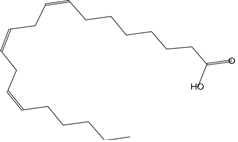 |
| 34 | trans-13-Octadecenoic  acid | 13.95 | C18H34O2 | 282 | 4.07 |  |
| 35 | Octadecanoic acid  (CAS) | 14.29 | C18H36O2 | 284 | 1.30 |  |
| 36 | 9-octadecenamide | 14.72 | C18H35NO | 281 | 0.53 | 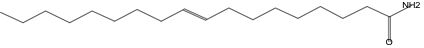 |
| 37 | 9-Octadecenoic acid  (Z)-, oxiranylmethyl  Ester | 14.84 | C21H38O3 | 338 | 0.05 |  |
| 38 | Cyclopentanetridecan  oic acid, methyl ester  (CAS) | 14.96 | C19H36O2 | 296 | 0.39 |  |
| 39 | Methyl arachidonate | 16.09 | C21H34O2 | 318 | 0.72 | 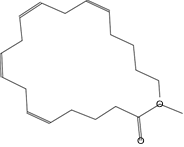 |
| 40 | Arachidonic acid | 16.97 | C20H32O2 | 304 | 1.07 | 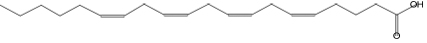 |
| 41 | cis-5,8,11,14,17-Eicosa  pentaenoic acid | 17.11 | C20H30O2 | 302 | 1.35 | 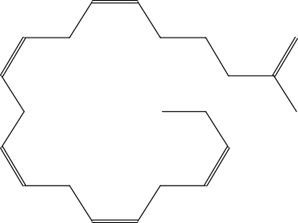 |
| 42 | Linolenic acid,  2-hydroxy-1-(hydroxy  methyl) ethyl ester  (Z,Z,Z)- | 20.07 | C21H36O4 | 352 | 0.13 |  |
| 43 | 9-Hexadecenoic acid | 20.41 | C16H30O2 | 254 | 0.30 |  |
| 44 | Hexadecanoic acid,  2-hydroxy-1-(hydroxym  ethyl) ethyl ester (CAS | 20.56 | C19H38O4 | 330 | 0.69 |  |
| 45 | Glycidyl palmitoleate | 20.75 | C19H34O3 | 310 | 0.08 |  |
| 46 | Hexadecanoic acid,  2,3-dihydroxypropyl  ester (CAS) | 20.90 | C19H38O4 | 330 | 0.13 | 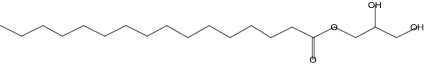 |
| 47 | Lucenin 2 | 21.98 | C27H30O16 | 610 | 0.08 | 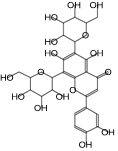 |
| 48 | cis-13-Eicosenoic acid | 22.41 | C20H38O2 | 310 | 0.27 | 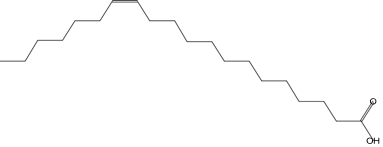 |
| 49 | Methyl arachidonate | 22.69 | C21H34O2 | 318 | 0.06 | 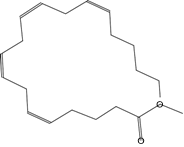 |
| 50 | cis-5,8,11,14,17-Eicosa  pentaenoic acid | 22.88 | C20H30O2 | 302 | 0.07 | 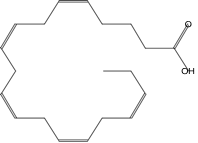 |
| 51 | Cholesterol margarate | 26.62 | C44H78O2 | 638 | 0.09 |  |
| 52 | 13-Docosenamide, (Z) | 26.92 | C22H43NO | 337 | 0.57 | 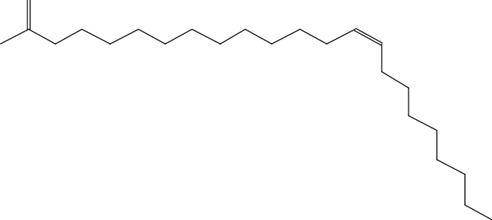 |
| 53 | 5,8,11,14-Eicosatetrae  noic acid, ethyl ester,  (all-Z)- | 27.82 | C22H36O2 | 332 | 0.30 |  |
| 54 | i-Propyl  5,8,11,14,17-eicosapen  taenoate | 28.02 | C23H36O2 | 344 | 0.17 |  |
| 55 | 2-[4-methyl-6-(2,6,6-tr  imethylcyclohex-1-eny  l)hexa-1,3,5-trienyl]cy  clohex-1-en-1-carboxa  ldehyde | 28.66 | C23H32O | 324 | 0.07 | 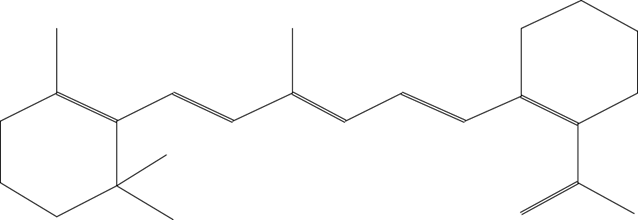 |
| 56 | Cholest-5-en-3-ol (3á)-  (CAS) | 34.06 | C27H46O | 386 | 18.73 | 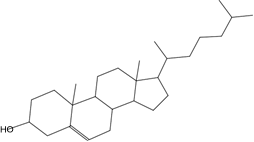 |
| 57 | Cholesta-3,5-dien-7-o  ne (CAS) | 35.80 | C27H42O | 382 | 0.21 | 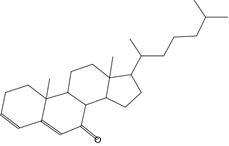 |
| 58 | Cholest-4-en-3-one  (CAS) | 36.60 | C27H44O | 384 | 0.95 | 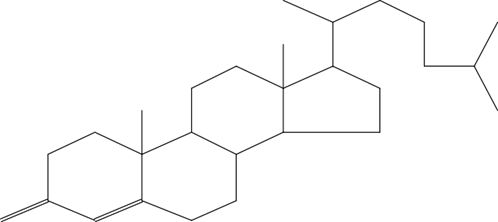 |
| 59 | 2,6-Di-t-butyl-4-[3-(2,3  -epoxypropylthio)propy  l]phenol | 43.28 | C20H32O2S | 336 | 1.74 | 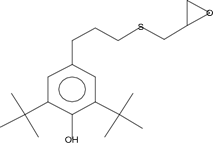 |
